# Comment on “Chronic kidney disease is associated with worse survival after revascularization in subjects with genetically diagnosed familial hypercholesterolemia and significant coronary artery disease”

**DOI:** 10.1016/j.ahjo.2026.100980

**Published:** 2026-10-21

**Authors:** Archana, Baskaran Kuppusamy, Paleri Madhumita, E. Prasanna Kumar

**Affiliations:** a Department of Pathology, Meenakshi Medical College Hospital & Research Institute, Meenakshi Academy of Higher Education and Research, Enathur, Kanchipuram, Tamil Nadu, 631552, India; b Central Research Laboratory, Meenakshi Medical College Hospital & Research Institute, Meenakshi Academy of Higher Education and Research, India; c Arulmigu Meenakshi College of Nursing, Meenakshi Academy of Higher Education and Research, India

**Keywords:** Chronic kidney disease, Familial hypercholesterolemia, Coronary artery disease, Revascularization, Survival, Estimated glomerular filtration rate, Cardiovascular outcomes, Percutaneous coronary intervention, Coronary artery bypass grafting, Risk factors

Dear Editor,

Liang et al. [1] followed 79 patients with confirmed familial hypercholesterolemia (FH) after coronary revascularization and concluded that chronic kidney disease (CKD) and lower estimated glomerular filtration rate (eGFR) were associated with worse survival. Outcome data in defined FH after revascularization are scarce and this carefully genotyped cohort is a useful one. We would however urge caution before CKD is read as a marker in this population.

The survival comparison is unadjusted. Patients who died were 15 years older at index admission than survivors (71.3 vs 56.2 years *p* < 0.001). Egfr declines with age. Neither the log-rank test nor the group comparisons can show whether CKD carries information beyond age [2]. With 11 deaths a full multivariable model is not possible. A Cox model containing only age and CKD or age and eGFR as a continuous term would be feasible and would show whether the association holds. As it stands the finding may largely restate that older patients die often [3]. Because the outcome was all-cause death it is also unclear whether CKD predicted deaths or mainly non-cardiac deaths in older patients.

The definition of CKD also needs clarification. The Methods require eGFR below 60 mL/min/1.73 m^2^ for least three months yet eGFR appears to come from the index admission. If a single measurement was used patients with contrast-related reductions around catheterization may have been classed as having CKD [4].

The time origin of follow-up raises a concern. Patients entered the cohort at a catheterization between 2010 and 2019. Survival is measured from revascularization and previous PCI or CABG was taken from their history. If some procedures predated the index admission those patients had to survive until enrollment to be included. That interval is time and patients who died soon after revascularization would be missing. Reporting how many procedures preceded the index admission or starting follow-up at enrollment would resolve this.

A similar problem affects the medication comparison. Statin use required at four weeks of prescriptions and it was lower among those who died (81.8% vs 98.5% *p* = 0.049). Patients who died sooner had time to accumulate prescriptions so this difference may reflect follow-up length rather than any protective effect of treatment.

The revascularization data are also difficult to interpret. Among those who died nine had PCI and six had CABG, a total exceeding the 11 deaths so some patients had both. Elsewhere 56 patients are described as receiving PCI. Testing overlapping categories as though they were groups leaves the comparison of strategies unclear. The higher proportion of CABG among deaths (54.5% vs 25.0% *p* = 0.071) is said to suggest disease burden yet Jeopardy scores did not differ (10.6, vs 9.3 *p* = 0.219).

The study adds welcome data on a group. An age-adjusted analysis, confirmation that kidney dysfunction was chronic and a defined time origin would make its main message considerably more convincing.

## CRediT authorship contribution statement

**Archana:** Writing – review & editing, Writing – original draft, Visualization. **Baskaran Kuppusamy:** Conceptualization, Formal analysis, Data curation. **Paleri Madhumita:** Writing – review & editing, Writing – original draft, Visualization. **E. Prasanna Kumar:** Writing – review & editing, Writing – original draft, Visualization.

## Informed consent

Not applicable.

## Ethical approval

Not applicable (Commentary on a previously published article).

## Declaration of Generative AI and AI-assisted technologies in the writing process

Generative AI tools were used only for language refinement and formatting assistance. All scientific interpretation, critical appraisal, and manuscript preparation were performed independently by the authors.

## Funding

None.

## Declaration of competing interest

The authors declare that there are no conflicts of interest.

## Data Availability

No new data were generated or analyzed in this study.
